# Multiplicative Effects of Social and Psychological Risk Factors on College Students’ Suicidal Behaviors

**DOI:** 10.3390/brainsci8050091

**Published:** 2018-05-17

**Authors:** Shervin Assari

**Affiliations:** 1 Department of Psychiatry, University of Michigan, Ann Arbor, MI 48109, USA; assari@umich.edu; Tel.: +1-734-647-7944; Fax: +1-734-763-7379; 2 Center for Research on Ethnicity, Culture and Health, School of Public Health, University of Michigan, Ann Arbor, MI 48109, USA

**Keywords:** college students, suicide, sexual orientation, depression, anxiety, substance use, abuse, violence

## Abstract

Less is known about the multiplicative effects of social and psychological risk and protective factors of suicidality on college campuses. The current study aimed to investigate the multiplicative effects of social (identifying oneself as gay/lesbian, financial difficulty, violence victimization, and religiosity) and psychological (anxiety, depression, problem alcohol use, drug use) and risk/protective factors on suicidal behaviors among college students in the United States. Using a cross-sectional design, the Healthy Mind Study (HMS; 2016–2017), is a national online survey of college students in the United States. Social (identifying oneself as gay/lesbian, violence victimization, financial difficulty, and religiosity) and psychological (anxiety, depression, problem alcohol use, and drug use) risk/protective factors were assessed among 27,961 individuals. Three aspects of suicidality, including ideation, plan, and attempt, were also assessed. Logistic regression models were used for data analysis. Financial difficulty, violence victimization, identifying oneself as gay/lesbian, anxiety, depression, and drug use increased, while religiosity reduced the odds of suicidal behaviors. Multiplicative effects were found between the following social and psychological risk factors: (1) financial difficulty and anxiety; (2) financial difficulty and depression; (3) depression and drug use; (4) problem alcohol use and drug use; and (5) depression and problem alcohol use. There is a considerable overlap in the social and psychological processes, such as financial stress, mood disorders, and substance use problems, on risk of suicide in college students. As social and psychological risk factors do not operate independently, comprehensive suicidal risk evaluations that simultaneously address multiple social and psychological risk factors may be superior to programs that only address a single risk factor.

## 1. Background

Suicide is the second leading cause of death for college students, second only to traffic injury [1]. The high risk of suicide in college students is also contributing to suicide as the third leading cause of death in youth and young adults between 15 and 24 years [2]. As a result, any epidemiological study that can provide additional insight into suicide prevention on college campuses is extremely valuable [3,4,5,6,7,8]. 

Transition to college is considerably stressful, and a large proportion of students feel lost, lonely, confused, anxious, inadequate, and stressed [1,9]. Given the associated stress, this transition is a risk factor for several suicide risk factors, such as depression [10,11], binge drinking [12], drug use [13], and violence victimization [14]. More than one-third of college students engage in binge drinking, and about 1 in 5 use illicit drugs [15]. The burden of substance and drug use at an early age is very considerable for youth, families, communities, and the nation [13].

Comorbidity is the rule, rather than the exception, when it comes to psychological risk factors of suicide. Psychiatric disorders including anxiety, depression, problem alcohol use, and drug use all tend to be comorbid. For instance, anxiety and depression are closely linked [16], and more than 50% of individuals who have depressive or anxiety disorder suffer from the other disorder [16]. Problem alcohol use and drug use are both also often comorbid [17]. Finally, a considerable proportion of individuals with affective or anxiety disorders also have substance use problems, also defined as dual diagnosis [18].

As suicide risk factors have common risk factors themselves, and tend to co-occur, some students are also at risk of multiple risk factors [19]. Most of the studies focusing on risk factors of suicide on college campuses have exclusively focused on the separate or additive effects of risk factors on suicidality [3,20,21,22,23,24], with very limited information being available regarding multiplicative effects of suicidal behaviors among university students [25,26,27].

Outside the context of college campuses, however, there is a growing literature that suggests psychological risk factors of suicide do not operate independently, but interact with each other [28,29,30]. To give an example, the effects of depression and anxiety may depend on substance use problems [29,30]. Religious involvement may also reduce the effects of psychiatric disorders on suicidality [28].

### Aim

The current study aimed to study the multiplicative effects of social (identifying oneself as gay/lesbian [31], financial difficulty [32], violence victimization [33], and religiosity [34]) and psychological (anxiety [35], depression [36], problem alcohol use [37], and drug use [38]) risk and protective factors, on suicidal behaviors in a national sample of college students in the United States. These social and psychiatric risk factors were extracted from a review of systemic reviews on risk and protective factors of suicidal behaviors [39]. We were specifically interested in the (1) interactions between substance use types; (2) interaction between substance use and affective disorders; (3) interaction between religion and psychiatric disorders; (4) interaction between gay/lesbian and psychiatric disorders; and (5) interaction between financial difficulty and psychiatric disorders. These were chosen based on an extensive literature review on risk factors of suicide.

## 2. Methods

### 2.1. Design and Setting

With a cross-sectional design, this study used data from the Healthy Mind Study (HMS), an online mental health survey of American college students. HMS is an annual web-based survey that examines mental wellbeing of undergraduate and graduate students in the United States. The study collects data on sociodemographic factors, mental health status, stigma, substance use, and service utilization [40,41]. Since launch in 2007, HMS has collected data from 150 colleges and universities, with over 175,000 survey respondents.

### 2.2. Ethics

The HMS fully protects the privacy of its participants and confidentiality of their data. The HMS protocol was approved by the University of Michigan Health Sciences and Behavioral Sciences Institutional Review Board (IRB). The study was covered by a Certificate of Confidentiality from the National Institutes of Health (NIH). All participants provided informed consent.

### 2.3. Sampling and Participants

Sampling within schools depend on school size (overall body of students). Participating schools provide a randomly selected sample of currently enrolled students who are at least 18 years old. Most large schools provide a random sample of 4000 students, while smaller schools use census (all students). Universities and schools with graduate students are asked to include both undergraduate and graduate students in the sample. As HMS is a web-based survey, selected students are invited and reminded to participate via emails.

### 2.4. Data Collection

As a web-based survey, HMS uses three standard survey modules on all participating campuses: demographics, mental health, and services utilization.

Data collection protocol begins with an email invitation. All the non-responders are contacted via email up to three times. Reminder emails are sent with 2–4 day intervals. Each email communication contains the URL to the electronic survey.

### 2.5. Measures

#### 2.5.1. Covariates

The study evaluated the following variables: race and ethnicity, parental socioeconomic status (SES), gender, age, sexual orientation, parental education, international status, graduate status, history of exposure to violence, and mental health (depression, general anxiety, and suicidality). Age was a continuous measure. 

Race and Ethnicity. Race and ethnicity were measured using a single item: “What is your race/ethnicity?” Participants were asked to select all that apply. The response options included (1) African American/Black; (2) American Indian or Alaskan Native; (3) Asian American/Asian; (4) Hispanic/Latino; (5) Native Hawaiian or Pacific Islander; (6) Middle Eastern, Arab, or Arab American; and (7) others. This variable was operationalized as two dichotomous variables (1 White, others 0), and (1 Hispanic/Latino, non-Hispanic/non-Latino 0).

#### 2.5.2. Independent Variables

Sexual Orientation. Sexual orientation was measured using the item “How would you describe your sexual orientation?” Responses included (1) heterosexual; (2) lesbian; (3) gay; (4) bisexual; (5) queer; (6) questioning; (7) others. We operationalized this variable as a dichotomous variable (1 heterosexual, 0 others).

Violence Victimization. The history of exposure to lifetime violence measured using three items: psychological, physical, and sexual violence. Items included (1) “Over the past 12 months, were you called names, yelled at, humiliated, judged, threatened, coerced, or controlled by another person?”; (2) “Over the past 12 months, were you kicked, slapped, punched or otherwise physically mistreated by another person?”; and (3) “In the past 12 months, has anyone had unwanted sexual contact with you?” Responses were (1) yes and (0) no. We operationalized violence victimization as a dichotomous variable, which reflected any exposure, regardless of its type. 

Religiosity. Religiosity was measured using a single item measure “How important is religion in your life?” This measure focuses on perceived religiosity. Responses included (5) very important; (4) important; (3) neutral; (2) unimportant; and (1) very unimportant. Single item measures are frequently used in large scale surveys to measure involvement in religion [42,43].

Financial Difficulty. Financial difficulty was measured using the following item: “How would you describe your financial situation right now?” The responses included (1) always stressful; (2) often stressful; (3) sometimes stressful; (4) rarely stressful; and (5) never stressful [3]. 

Anxiety. General anxiety was measured using the 7-item Generalized Anxiety Disorder (GAD-7) scale [44], a self-report questionnaire designed to identify probable cases of generalized anxiety disorder. GAD-7 measures symptom severity over the past 2 weeks [44,45] by asking participants how frequently, during the last 2 weeks, have they experienced seven core symptoms of generalized anxiety disorder. Item responses range from “not at all”, (0) to “nearly every day”, (3). A total score is calculated, with a higher score reflecting higher symptoms. This measure has shown high reliability, construct validity, and factorial validity in the general population and clinical sample [46]. GAD-7 is designed based on the DSM-IV diagnostic criteria. 

Depression. Depression was measured using the Patient Health Questionnaire (PHQ-9). In line with the Composite International Diagnostic Interview Short-Form [47], participants were asked to think about the 2-week period with the highest symptom levels: “Think about the two-week period in the past year when you experienced the two problems below the most frequently. During that period, how often were you bothered by these problems?” We then listed the PHQ items, such as “little interest or pleasure in doing things” and “feeling down, depressed, or hopeless”. Item responses used a four-level category ranging from 0 (none) to 3 (nearly every day) points per item, with a higher score reflecting greater symptom frequency [46].

Problem Alcohol Use. Problem alcohol use was measured using the following three items: (1) “How often do you have a drink containing alcohol?”; (2) “How many drinks containing alcohol do you have on a typical day when you are drinking?”, and “How often do you have 4 (female)/5 (male)/4 or 5 (not female or male) or more drinks on 1 occasion? (1 drink is a can of beer, a glass of wine, a wine cooler, a shot of liquor, or a mixed drink)”. The response items for the first item included (1) never; (2) monthly or less; (3) 2–4 times a month; (4) 2–3 times a week, and (5) 4 or more times a week. The responses to the second question included 1 = 1 or 2, 2 = 3 or 4, 3 = 5 or 6, 4 = 7 to 9, 5 = 10 or more. The responses to the last item were (1) never; (2) less than monthly; (3) monthly; (4) weekly; and (5) daily or almost daily. These items were drawn from the AUDIT measure developed by Saunders et al., in 1993, and provided Problem alcohol use was measured using the following three items, based on the definition of problem alcohol use by the National Institute on Alcohol Abuse and Alcoholism (NIAAA) [48].

Drug Use. Drug use was measured using the following item: “Over the past 30 days, have you used any of the following drugs?” Participants were asked to select all that apply from the following list: (1) marijuana; (2) cocaine (any form, including crack, powder, or freebase); (3) heroin; (4) methamphetamines (also known as speed, crystal meth, or ice); (5) other stimulants (such as Ritalin, Adderall) without a prescription; (6) ecstasy, and 7 = other drugs without a prescription [49].

#### 2.5.3. Dependent Variables

Suicidality. Suicidal behaviors were measured with three items: suicidal ideation, suicidal plan, and suicidal attempt. Each of these items was a yes/no question taken from the National Comorbidity Survey (NCS). The questions were “In the past year, did you ever seriously think about committing suicide?” “In the past year, did you make a plan for committing suicide?” and “In the past year, did you attempt suicide?” The last two questions were only asked if the responses to the first item was “yes” [50]. 

### 2.6. Data Analysis

We used SPSS 24.00 (SPSS Inc., Chicago, IL, USA) for data analysis. For descriptive purposes, we reported frequency tables (%) and means (SD). Spearman’s correlation test was used for bivariate analysis. We ran multiple logistic regression models for data analysis. The independent variables in this study were depression, anxiety, problem alcohol use, drug use, identifying oneself as gay/lesbian, violence victimization, and religiosity. The dependent variable was suicidality. Race, age, gender, parental education, transfer status, enrolment status, international status, and academic level were covariates. Models tested two by two interaction terms between the independent variables, according to our hypothesis (rather than fully exploratory). From our logistic regression models, we reported odds ratio (OR), 95% CI, and *p*-values. *p*-values less than 0.05 were considered statistically significant.

## 3. Results

The current study included 27,961 individuals. Descriptive statistics are shown in Table 1. About 12%, 5%, and 1% of the students reported suicidal ideation, plan, and attempt, respectively, over the past 12 months.

Table 2 describes the correlations between study variables. Financial difficulty, violence victimization, gay/lesbian status, anxiety, depression, and drug use were correlated with suicidality.

Table 3 shows the results of three logistic regression models with suicide ideation, suicide plan, and suicide attempt as outcomes. These models showed that financial difficulty, anxiety, depression, drug use, identifying oneself as gay/lesbian, and violence victimization increase the risk of suicidal behaviors over the past 12 months. Higher religiosity was associated with lower odds of suicidal ideation. Problem alcohol use, however, was not associated with suicidal outcomes over the past 12 months.

Table 4 reports the results of the interactions between financial stress and anxiety and depression. The models included all the main effects in addition to two interaction terms: (1) financial stress × anxiety; and (2) financial stress × depression. A synergistic effect was found between financial stress and anxiety. Multiplicative effects were found between depression and financial stress.

Table 5 tested the interactions between drug use and alcohol, depression, and anxiety. Multiplicative (subadditive) effects were found between drugs and alcohol, and drugs and depression. Religiosity did not interact with other suicide risk factors (interactions tested but not shown as they were not significant).

## 4. Discussion

In a national sample of college students in the United States, the current study showed two major findings. First, a wide range of social (identifying oneself as gay/lesbian, violence victimization, and religiosity) and psychological (anxiety, depression, problem alcohol use, and drug use) risk/protective factors are associated with odds of suicidality. Furthermore, the effects of social and psychological risk/protective factors are multiplicative (subadditive) rather than additive or synergistic. Subadditive effects were found between the following risk factor pairs: (1) financial difficulty and anxiety; (2) financial difficulty and depression; (3) depression and drug use; (4) problem alcohol use and drug use; and (5) depression and problem alcohol use. 

Subadditive effects of social and psychological risk factors can be attributed to similarities and common elements that various psychiatric disorders share. Stress is a common cause for depression, anxiety, substance use, and suicide [51,52]. Dysregulation of the hypothalamus–pituitary–adrenal (HPA) axis is commonly seen in patients with anxiety, depression, and other mental conditions [53]. Thus, we should expect that some of the effects of social risk factors on suicide to be through psychiatric disorders [54], such as anxiety, depression, drug use, problem alcohol use, and depression [55].

Several psychological risk factors increase the risk of suicide through common mechanisms, and suicidality is a common element of all these conditions. “Emotional dysregulation” is a common element of various internalizing and externalizing disorders, such as anxiety and depression [56]. Poor social relation and loneliness are also commonly seen across various disorders, such as anxiety, depression, and substance use disorder [57]. Substance use may also be a presentation of anxiety and depression, as individuals with affective disorders are at a higher risk for self-medication using drugs and alcohol, to reduce their psychological pain [58]. Various psychiatric disorders have common genetic predispositions [59,60]. The serotonin transporter gene (5-HTT) variant, for example, influences several psychiatric disorders [61]. Certain alleles of the dopamine D2 receptor gene also increase the risk of various forms of substance use, such as alcohol and drugs [62].

The overlap and comorbidities between suicide risk factors may be due to the co-occurrence, either sequentially or simultaneously, by coincidence, or causal link between the two [63,64]. For instance, while anxiety and depression share common social and genetic risk factors [65], baseline anxiety increases subsequent risk of depression [66], and depression increases the future risk of substance use [67].

The current results on subadditive effects of risk factors may be also due to the common biological changes and physiopathologies that stress, anxiety, and depression share. Such overlap may be because stress, anxiety, and depression all cause impaired neuroplasticity, impact neurotrophic factors, and result in overlapping structural and functional changes in brain [68,69]. Chronic stress, financial hardship, anxiety, and depression are all associated with structural brain changes, such as a loss of dendritic spines and synapses, reduced dendritic arborization, as well as reduced glial cells in the hippocampus [70,71,72,73]. Dysregulation of the hypothalamus–pituitary–adrenal (HPA) axis, which has been observed in several psychiatric conditions, and suicidality, may also explain the subadditive effects of risk factors [74]. All of these shared brain changes may explain why multiple social and psychological risk factors have subadditive effects.

Although this study did not explore race and ethnic variation in the multiplicative effects of risk and protective factors on suicidal ideation, some research suggests that whether the effects of psychiatric disorders on suicide are subadditive or synergistic depends on race and ethnicity. Among Whites, for example, multiple psychological risk factors seem to have subadditive effects on suicide [75,76,77], while the same effects seem to be synergistic for Blacks [29,30]. Future research should explore racial and ethnic variation in the effects of risk and protective factors on suicide. Gender may also alter the relevance of multiple risk factors on suicidal behaviors. 

### 4.1. Implications

An understanding of comorbidity between various social and psychological risk factors of suicide is essential in developing effective protocols and guidelines for screening, diagnosis, and treatment of suicide. Unfortunately, whether multiple suicide risk factors have additive, synergistic, or subadditive effects on suicide among college students, and whether these patterns themselves depend on race, gender, and other demographic factors, is still unknown. Thus, it is essential to investigate the non-linear and complex patterns by which multiple risk factors impact risk of suicidal behaviors on college campuses.

### 4.2. Limitations

The current study had a few limitations. Suicidal behaviors were measured using three single item measures. Although this is a common practice in epidemiological studies of suicide risk, future research should replicate these findings using more detailed measures of suicide. Columbia suicide scale and other standardized well-validated measures may provide more detailed information on the same outcome. Another limitation was that the study did not use a structured diagnostic interview, but symptoms of anxiety and depression. In addition, we did not measure substance use disorder, but problem drinking. Finally, some potential confounders, such as family income, history of psychotropic medications, and familial mental health history were omitted. Hopelessness is a very important and independent risk factor for suicide, particularly amongst youth [78]. Future research is needed to replicate these findings in future. 

## 5. Conclusions

To summarize the results, there is a considerable overlap in the social and psychological processes, such as financial difficulty, anxiety, depression, problem alcohol use, and drug use, which increase the risk of suicide among college students. The take-home message of this study is that the social and psychological risk factors do not operate independently but their effects depend on the presence or absence of other risk and protective factors. The results advocate for comprehensive suicide prevention programs that simultaneously address a wide range of social and psychological risk and protective factors on college campuses. Future research should test whether such a comprehensive approach will be superior to the programs that only address a single risk factor.

## Figures and Tables

**Table 1:** Descriptive characteristics in the participants.

	*N*	%
Gender		
Male	9582	34.3
Female	18,353	65.7
Race		
Others	7184	25.7
White	20,772	74.3
Ethnicity		
Hispanic	25,818	92.34
Non-Hispanic	2143	7.66
Level		
Undergraduate	21,789	80.65
Graduate	5227	19.35
Enrolment		
Full-time student	24,963	90.11
Part-time student	2741	9.89
Transferred		
No	14,363	75.71
Yes	4607	24.29
Anxiety		
No	24,842	88.85
Yes	3119	11.15
Depression		
No	20,238	72.38
Yes	7723	27.62
Drug Use		
No	20,882	74.68
Yes	7079	25.32
Problem Alcohol Use		
No	16,657	59.57
Yes	11,304	40.43
Gay/Lesbian		
No	23,371	83.58
Yes	4590	16.42
Violence Victimization		
No	23,553	84.24
Yes	4408	15.76
Suicidal Ideation		
No	24,606	88.23
Yes	3282	11.77
Suicidal Plan		
No	26,532	95.29
Yes	1312	4.71
Suicidal Attempt		
No	27,542	98.95
Yes	292	1.05

**Table 2:** Correlation matrix between variables.

	1	2	3	4	5	6	7	8	9	10	11	12	13	14	15	16	17	18	19
1 Age	1	−0.02 **	−0.06 **	−0.03 **	−0.33 **	0.32 **	0.39 **	−0.18 **	0.09 **	−0.04 **	−0.06 **	−0.11 **	−0.04 **	−0.05 **	−0.04 **	0.08 **	−0.06 **	−0.05 **	−0.02 **
2 Gender (Female)		1	−0.00	0.01 *	0.01	0.00	−0.01	−0.05 **	0.10 **	0.07 **	0.06 **	−0.07 **	0.60 **	−0.01	0.12 **	0.08 **	−0.01	−0.01	0.01
3 Race (White)			1	−0.24 **	0.06 **	0.01 *	−0.04 **	0.15 **	−0.03 **	0.03 **	0.00	0.07 **	0.11 **	0.01 *	0.02 **	−0.14 **	0.01	0.01	−0.02 **
4 Ethnicity (Hispanic)				1	0.03 **	0.01	0.00	−0.13 **	0.05 **	0.00	0.03 **	0.03 **	0.01	0.04 **	0.01	0.01	0.02 **	0.01 *	0.01
5 Degree (Undergraduate)					1	−0.03 **	0.03 **	−0.13 **	0.04 **	0.06 **	0.11 **	0.09 **	−0.02 **	0.05 **	0.09 **	0.00	0.09 **	0.06 **	0.03 **
6 Enrolment Status						1	0.15 **	−0.14 **	0.05 **	0.01	0.00	−0.02 **	−0.02 **	−0.01	0.02 *	0.03 **	−0.00	−0.01	0.00
7 Transfer Status							1	−0.18 **	0.14 **	0.03 **	0.03 **	−0.04 **	−0.03 **	−0.03 **	0.01	0.03 **	−0.01	−0.02 **	−0.01
8 Parental Education								1	−0.26 **	−0.05 **	−0.08 **	0.05 **	0.06 **	0.01	−0.06 **	−0.07 **	−0.03 **	−0.02 **	−0.02 **
9 Financial Difficulty									1	0.20 **	0.27 **	0.05 **	0.05 **	0.07 **	0.17 **	0.03 **	0.14 **	0.10 **	0.06 **
10 Anxiety										1	0.45 **	0.07 **	0.05 **	0.12 **	0.17 **	−0.04 **	0.24 **	0.18 **	0.10 **
11 Depression											1	0.11 **	0.04 **	0.18 **	0.23 **	−0.09 **	0.35 **	0.25 **	0.12 **
12 Drug Use												1	0.11 **	0.11 **	0.09 **	−0.18 **	0.11 **	0.07 **	0.05 **
13 Problem Drinking													1	0.01 *	0.09 **	−0.05 **	0.00	−0.00	0.00
14 Gay/Lesbian														1	0.13 **	−0.18 **	0.19 **	0.15 **	0.09 **
15 Violence Victimization															1	−0.01 *	0.21 **	0.16 **	0.11 **
16 Religion Involvement																1	−0.08 **	−0.04 **	−0.02 **
17 Suicide Ideation																	1	0.61 **	0.28 **
18 Suicide Attempt																		1	0.37 **
19 Suicide Plan																			1

* *p* < 0.05 (2-tailed); ** *p* < 0.01 (2-tailed).

**Table 3:** Summary of three logistic regression models with suicidal behaviors as outcomes.

	Ideation		Plan		Attempt	
	OR	95% CI	OR	95% CI	OR	95% CI
Age	0.97 ***	0.96–0.99	0.98 *	0.96–1.00	0.99	0.96–1.03
Gender (Female)	0.73 ***	0.64–0.84	0.75 **	0.62–0.90	0.87	0.59–1.28
Race (White)	0.96	0.85–1.08	0.96	0.81–1.13	0.58 ***	0.43–0.79
Ethnicity (Hispanic)	1.08	0.91–1.28	1.12	0.88–1.42	0.81	0.50–1.31
Undergraduate Degree	2.05 *	1.03–4.07	1.91	0.69–5.35	0.77	0.18–3.27
Enrollment Status	1.23 #	0.99–1.53	1.26	0.93–1.71	1.44	0.82–2.53
Transfer Student	0.91	0.81–1.03	0.78 **	0.65–0.93	0.70 #	0.48–1.02
Parent Education	1.04 *	1.01–1.08	1.02	0.97–1.07	0.99	0.90–1.09
Financial Stress	1.15 ***	1.10–1.21	1.11 **	1.04–1.19	1.22 **	1.06–1.40
Anxiety	1.62 ***	1.44–1.82	1.66 ***	1.42–1.94	1.40 *	1.04–1.90
Depression	5.10 ***	4.58–5.69	5.76 ***	4.85–6.85	5.65 ***	3.85–8.30
Drug Use	1.35 ***	1.22–1.50	1.24 **	1.07–1.43	1.51 **	1.13–2.01
Problem Alcohol Use	1.03	0.90–1.16	0.91	0.76–1.09	1.00	0.70–1.43
Gay/Lesbian	1.88 ***	1.69–2.10	2.05 ***	1.77–2.37	2.48 ***	1.86–3.30
Abuse Victimization	2.16 ***	1.94–2.40	2.16 ***	1.87–2.50	2.75 ***	2.06–3.65
Religion Involvement	0.93 ***	0.89–0.96	0.97	0.92–1.02	0.93	0.84–1.04

# *p* < 0.1 (2-tailed); * *p* < 0.05 (2-tailed); ** *p* < 0.01 (2-tailed); *** *p* < 0.001 (2-tailed).

**Table 4:** Summary of three logistic regressions with suicidal behaviors as outcomes.

	Ideation		Plan		Attempt	
	OR	95% CI	OR	95% CI	OR	95% CI
Age	0.97 ***	0.96–0.99	0.98 *	0.96–1.00	0.99	0.96–1.03
Gender (Female)	0.73 ***	0.64–0.84	0.75 **	0.62–0.90	0.86	0.59–1.27
Race (White)	0.96	0.86–1.08	0.96	0.81–1.14	0.58 ***	0.43–0.79
Ethnicity (Hispanic)	1.09	0.91–1.29	1.13	0.89–1.44	0.80	0.49–1.30
Undergraduate Degree	2.06 *	1.04–4.09	1.92	0.69–5.35	0.77	0.18–3.24
Enrollment Status	1.23 #	0.99–1.52	1.25	0.92–1.70	1.45	0.82–2.54
Transfer Student	0.91	0.81–1.03	0.78 **	0.65–0.93	0.70 #	0.48–1.01
Parent Education	1.04 *	1.01–1.08	1.02	0.97–1.07	0.99	0.90–1.09
Financial stress	1.24 ***	1.15–1.34	1.32 ***	1.16–1.50	1.28	0.95–1.72
Anxiety	1.20	0.87–1.65	1.30	0.86–1.99	2.36 #	0.97–5.74
Depression	7.63 ***	5.91–9.86	10.95 ***	7.28–16.47	5.17 ***	2.00–13.38
Drug Use	1.35 ***	1.22–1.50	1.24 **	1.08–1.44	1.51 **	1.13–2.01
Problem Alcohol Use	1.02	0.90–1.16	0.91	0.76–1.09	1.00	0.70–1.43
Gay/Lesbian	1.88 ***	1.69–2.09	2.04 ***	1.76–2.36	2.48 ***	1.86–3.30
Abuse Victimization	2.16 ***	1.94–2.40	2.16 ***	1.87–2.50	2.73 ***	2.06–3.64
Religion Involvement	0.93 ***	0.89–0.96	0.97	0.92–1.02	0.93	0.84–1.04
Financial stress × Anxiety	1.12 *	1.00–1.25	1.10	0.95–1.27	0.84	0.63–1.12
Financial stress × Depression	0.84 ***	0.77–0.93	0.76 ***	0.65–0.89	1.03	0.72–1.46

# *p* < 0.1 (2-tailed); * *p* < 0.05 (2-tailed); ** *p* < 0.01 (2-tailed); *** *p* < 0.001 (2-tailed).

**Table 5:** Summary of three logistic regressions with suicidal behaviors as outcomes.

	Ideation		Plan		Attempt	
	OR	95% CI	OR	95% CI	OR	95% CI
Age	0.97 ***	0.96–0.99	0.98 *	0.96–1.00	0.99	0.96–1.03
Gender (Female)	0.74 ***	0.65–0.85	0.77 **	0.64–0.93	0.90	0.61–1.33
Race (White)	0.96	0.85–1.08	0.95	0.80–1.12	0.58 ***	0.43–0.79
Ethnicity (Hispanic)	1.08	0.91–1.28	1.12	0.88–1.43	0.81	0.50–1.31
Undergraduate degree	2.06 *	1.04–4.08	1.93	0.69–5.38	0.77	0.18–3.26
Enrollment status	1.23 #	0.99–1.53	1.26	0.93–1.71	1.44	0.82–2.53
Transfer	0.91	0.81–1.03	0.78 **	0.65–0.93	0.70 #	0.48–1.01
Parent Education	1.04 *	1.01–1.08	1.02	0.97–1.07	0.99	0.90–1.09
Financial stress	1.15 ***	1.10–1.21	1.11 **	1.04–1.19	1.22 **	1.06–1.40
Anxiety	1.57 ***	1.35–1.82	1.56 ***	1.28–1.91	1.38	0.91–2.08
Depression	5.52 ***	4.81–6.32	6.69 ***	5.39–8.31	6.77 ***	4.05–11.32
Drug Use	1.60 ***	1.33–1.93	1.79 ***	1.32–2.42	2.33 *	1.17–4.63
Problem Alcohol Drinking	1.07	0.92–1.23	1.00	0.81–1.22	1.10	0.72–1.67
Gay/Lesbian	1.89 ***	1.70–2.10	2.06 ***	1.78–2.38	2.49 ***	1.87–3.32
Abuse Victimization	2.16 ***	1.94–2.40	2.17 ***	1.87–2.51	2.74 ***	2.06–3.65
Religion Involvement	0.93 ***	0.89–0.96	0.97	0.92–1.02	0.94	0.84–1.04
Drugs × Depression	0.81 #	0.65–1.01	0.67 *	0.48–0.94	0.66	0.31–1.38
Drugs × Anxiety	1.08	0.85–1.38	1.17	0.86–1.60	1.04	0.57–1.88
Drugs × Alcohol	0.89	0.73–1.09	0.77 #	0.58–1.03	0.80	0.45–1.40

# *p* < 0.1 (2-tailed); * *p* < 0.05 (2-tailed); ** *p* < 0.01 (2-tailed); *** *p* < 0.001 (2-tailed).
